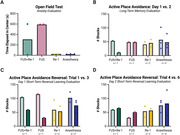# Preliminary Evidence for the Combined Efficacy of Focused Ultrasound Blood‐Brain Barrier Opening and Delivery of a Novel Anti‐Amyloid Re Complex for Memory Improvement in a 3xTg‐Alzheimer’s Disease Mouse Model

**DOI:** 10.1002/alz.090917

**Published:** 2025-01-09

**Authors:** Rebecca L Noel, Samantha L Gorman, Alec L Batts, Deny L Tsakris, Daniella L Jimenez, Maria Pelecanou, Marina L Sagnou, Elisa Konofagou

**Affiliations:** ^1^ Columbia University, New York, NY USA; ^2^ National Center for Scientific Research “Demokritos”, Agia Paraskevi, Athens Greece

## Abstract

**Background:**

Focused Ultrasound‐induced Blood‐Brain Barrier Opening (FUS‐BBBO) has demonstrated preventative and therapeutic efficacy for improving cognitive and pathological decline in Alzheimer’s Disease (AD). Previous work has demonstrated highly specific binding of a novel Re complex (Re‐1) complex to amyloid‐β (Aβ) *in vitro*, subsequently inhibiting fibril formation and reducing Aβ‐induced cytotoxicity in neuronal cell cultures. The aim of this preliminary study is to evaluate the efficacy of early intervention combining FUS‐BBBO and Re‐1 for anxiety amelioration and memory improvement in a triple transgenic (3xTg)‐AD mouse model.

**Method:**

Four‐month‐old, male, triple transgenic (3xTg)‐AD mice, were divided into four groups for the present study: FUS+Re‐1, FUS, Re‐1, and anesthesia sham. This study consists of ten, bi‐weekly treatments spanning five months. Following treatment, the animals underwent five days of behavioral testing for anxiety (Open Field Test, OFT), spatial memory (Active Place Avoidance, APA), and reversal learning (Active Place Avoidance Reversal) to evaluate the progression of their AD‐associated cognitive deficits.

**Result:**

Here we report that the combination of FUS‐BBBO and Re‐1 delivery improves anxiety, long‐term spatial memory, and short‐term reversal learning, compared to FUS or Re‐1 alone, and compared to the anesthesia sham group. The amount of time elapsed in the center of the brightly lit OFT arena is inversely related to each animal’s level of anxiety; thus, the FUS+Re‐1 cohort and the FUS cohorts exhibited the lowest levels of anxiety across the four groups (Figure 1A). Next, the number of shocks delivered to each animal over the course of an Active APA learning trial is inversely related to that animal’s spatial memory capacity. Comparison of animal performance in the final trial of the first day of training, with the first trial of the subsequent day demonstrates that the FUS+Re‐1cohort had the best long‐term memory of the four cohorts (Figure 1B). The FUS+Re‐1 group also demonstrated the most consistent reversal learning improvement when comparing the first and third (final) trial on each of the two APA reversal trial days (Figure 1C‐D).

**Conclusion:**

This promising preliminary study offers evidence in support of early intervention with amyloid‐targeting Re‐1 and FUS‐BBBO for cognitive improvement in AD.